# Hereditary Disease

**Published:** 1883-02

**Authors:** 


### HEREDITARY DISEASE.

There is, strictly speaking, no such thing. Children are not born diseased, however (some specific maladies excepted) much one or both parents are, but they are simply born with a predisposition to such parental malady. They are born with the material, with the powder, but actual disease will no more occur unless exciting causes are applied, than powder would detonate without the aid of fire. The observant reader has often felt surprised at seeing robust, hearty children, of parents who were seemingly at not a great remove from the grave; and if rational care were taken of such children, they would live to become healthy men and women. The practical lesson should be, a hopeful diligence in the rearing of children of diseased parentage. The difference between the children of healthy and diseased parents amounts to this: as to the latter, the powder is drier, they have less capability of resisting the causes of disease, the consequence is, a greater necessity for carefulness; this necessity is often felt, and practically attended to; the result is, that such persons are found living, scores of years after they have mouldered in the grave, who, in priding themselves on having constitutions which nothing could hurt, could not be made to feel the need of carefulness, and, consequently, perished long before their prime.

